# APICAL SPIKELET ABORTION (ASA) Controls Apical Panicle Development in Rice by Regulating Salicylic Acid Biosynthesis

**DOI:** 10.3389/fpls.2021.636877

**Published:** 2021-02-25

**Authors:** Dan Zhou, Weifeng Shen, Yuchao Cui, Yuqin Liu, Xijun Zheng, Yan Li, Minliang Wu, Shanru Fang, Chunhong Liu, Ming Tang, Yin Yi, Mingfu Zhao, Liang Chen

**Affiliations:** ^1^Xiamen Key Laboratory for Plant Genetics, School of Life Sciences, Xiamen University, Xiamen, China; ^2^Rice Research Institute, Fujian Academy of Agricultural Science, Fuzhou, China; ^3^Key Laboratory of State Forestry Administration on Biodiversity Conservation in Karst Area of Southwestern, School of Life Sciences, Guizhou Normal University, Guiyang, China; ^4^Key Laboratory of Plant Physiology and Developmental Regulation, School of Life Sciences, Guizhou Normal University, Guiyang, China

**Keywords:** apical panicle abortion, anther, *Oryza sativa*, reactive oxygen species, salicylic acid

## Abstract

Panicle degradation causes severe yield reduction in rice. There are two main types of panicle degradation: apical spikelet abortion and basal degeneration. In this study, we isolated and characterized the apical panicle abortion mutant *apical spikelet abortion* (*asa*), which exhibits degeneration and defects in the apical spikelets. This mutant had a pleiotropic phenotype, characterized by reduced plant height, increased tiller number, and decreased pollen fertility. Map-based cloning revealed that *OsASA* encodes a boric acid channel protein that showed the highest expression in the inflorescence, peduncle, and anther. RNA-seq analysis of the *asa* mutant vs wild-type (WT) plants revealed that biological processes related to reactive oxygen species (ROS) homeostasis and salicylic acid (SA) metabolism were significantly affected. Furthermore, the *asa* mutants had an increased SA level and H_2_O_2_ accumulation in the young panicles compared to the WT plants. Moreover, the SA level and the expression of *OsPAL3*, *OsPAL4*, and *OsPAL6* genes (related to SA biosynthesis) were significantly increased under boron-deficient conditions in the *asa* mutant and in *OsASA-*knockout plants. Collectively, these results suggest that the boron distribution maintained by *OsASA* is required for normal panicle development in a process that involves modulating ROS homeostasis and SA biosynthesis.

## Introduction

Rice (*Oryza sativa*), one of the most important food crops in the world, is the staple food of half the world’s population. The rice yield is mainly a result of the panicle number per unit area, grains per panicle, and grain weight, which are influenced by plant type, panicle type, and grain development (Xing and Zhang, 2010). Regarding one of the factors that affect the rice yield, panicle formation involves complex physiological and biochemical processes, including axillary meristem development, inflorescence establishment, and grain development. Many important genes related to panicle development have been reported, involving the regulation of axillary meristem initiation, spatiotemporal transformation related to meristem development, branch elongation, and the inflorescence structure (Teo et al., 2014). The *aberrant panicle organization 1* (*apo1*) mutant exhibits altered inflorescence architecture and changes in the control of floral organ identity, with a severely reduced number of spikelets and grains; in contrast, *APO1* overexpression increases the inflorescence branches and spikelets (Ikeda et al., 2005, 2007; Ikeda-Kawakatsu et al., 2009). The *IPA1* quantitative trait locus (QTL) encodes SQUAMOSA PROMOTER-BINDING PROTEIN-LIKE 14 (OsSPL14) and is regulated by the microRNA OsmiR156 *in vivo*; higher expression of *OsSPL14* in rice in the reproductive stage promotes panicle branching, increases the grain yield, accelerates flowering, and decreases the tiller number (Jiao et al., 2010; Miura et al., 2010; Luo et al., 2012). The *larger panicle* (*lp*) mutant has significantly increased panicle size, improved plant architecture, more inflorescence branches (especially primary branches), and more grains per panicle (Li et al., 2011). The *lax1* mutant exhibits severely reduced initiation and maintenance of rachis-branches, lateral spikelets, and terminal spikelets (Komatsu et al., 2001). Additionally, *LAX2* regulates the branching of the aboveground parts of rice except for the primary panicle branch; together, *LAX2* and *LAX1* regulate the process of axillary meristem formation (Tabuchi et al., 2011). In the rice *frizzy panicle* (*fzp*) mutant, floret formation is replaced by sequential rounds of branching; *FZP* (the ortholog of the maize *BD1* gene) is required to prevent axillary meristem formation within the spikelet meristem and permit the subsequent establishment of floral meristem identity (Komatsu et al., 2001, 2003). Lastly, a gain-of-function *DENSE AND ERECT PANICLE 1* (*DEP1*) mutation has been reported to enhance meristematic activity, reduce the inflorescence internode length, and increase the number of both primary and secondary panicle branches (Huang et al., 2009).

Panicle degradation reduces the yield per plant, seriously affecting rice production. There are two main types: apical spikelet abortion and basal degeneration. Temperature, humidity, and other environmental factors during panicle differentiation affect the degeneration degree, causing difficulties in genetic and biomolecular research on panicle degradation. Several QTLs related to panicle spikelet abortion have been reported. On chromosomes 1, 10, and 11, there are three main-effect QTLs underlying floret abortion of the rice panicle before flowering (Yamagishi et al., 2004). *SHORT PANICLE 1* (*SP1*), which encodes a peptide transporter (PTR) family member, regulates panicle branch elongation and is related to basal degeneration, with the *sp1* mutant exhibiting degenerated basal branches and spikelets (Li et al., 2009). Apical spikelet abortion is more prevalent than basal degeneration in rice production. Several QTLs related to spikelet degeneration have been identified (Tan et al., 2011). The *panicle apical abortion 8* (*pap8*) mutant exhibits degenerated spikelets that are associated with excess H_2_O_2_ accumulation (Cheng et al., 2011). The rice panicle degenerative mutant *tutou1* (*tut1*) exhibits severe defects; with apical panicle degeneration, TUT1 can activate the protein complex actin-related protein 2/3 to promote actin nucleation and polymerization *in vitro* (Bai et al., 2015). Mutation of *OsALMT7*, which encodes a putative aluminum-activated malate transporter, leads to programmed cell death in apical spikelets and spikelet degeneration in the apical portion of panicles (Heng et al., 2018). *SQUAMOSA PROMOTER-BINDING PROTEIN-LIKE 6* (*SPL6*) acts as a negative regulator of the inositol-requiring enzyme 1 (IRE1)-mediated endoplasmic reticulum (ER) stress signaling outputs, so that the *spl6-1* mutant with *IRE1* hyperactivation exhibits apical panicle abortion (Wang Q. L. et al., 2018). Knockout of *CALCINEURIN B-LIKE PROTEIN-INTERACTING PROTEIN KINASE 31* (*OsCIPK31*) causes a pleiotropic phenotype that includes apical panicle abortion (Peng et al., 2018). Lastly, *DEGENERATED PANICLE AND PARTIAL STERILITY 1* (*DPS1*) plays a vital role in regulating reactive oxygen species (ROS) homeostasis and anther cuticle formation, with the *dps1* mutant displaying apical panicle degeneration and reduced fertility (Zafar et al., 2019).

Salicylic acid (SA) is a key plant defense hormone with critical roles in various aspects of plant immunity (Zhang and Li, 2019). It also plays an important role in the regulation of plant growth, development, and responses to abiotic stresses (Hara et al., 2012). In higher plants, SA biosynthesis involves two distinct and compartmentalized pathways with multiple steps: the isochorismate synthase and phenylalanine ammonia-lyase pathways (Dempsey et al., 2011). ROS are crucial regulators of metabolism and plant responses to biotic and abiotic environmental stimuli, and ROS are also important for plant development (Waszczak et al., 2018). SA maintains root meristem activity in rice by promoting ROS accumulation (Xu et al., 2017). Additionally, WRKY75, SA, and ROS form a tripartite amplification loop to accelerate leaf senescence (Guo et al., 2017).

Boron (B) is an essential micronutrient for plant growth and development. Its principal known function in vascular plants is to maintain cell wall structure by cross-linking the pectic polysaccharide rhamnogalacturonan II (RG-II) (Kobayashi et al., 1996; Matoh et al., 1996; O’Neill et al., 2004). Boron is required mainly in developing tissues rather than mature tissues, so boron deficiency primarily restrains developing tissues, inhibiting root elongation, leaf expansion, and inflorescence development; this results in lack of pollen fertility, severe plant growth defects, and crop yield losses (Shorrocks, 1997; Lordkaew et al., 2011; Durbak et al., 2014; Routray et al., 2018).

In this study, we isolated and characterized an apical panicle abortion mutant, designated the *apical spikelet abortion* (*asa*) mutant, that exhibited degeneration of the top spikelets in the early stage of panicle development. Map-based cloning indicated that the gene responsible for apical spikelet abortion in the *asa* mutant, *OsASA* (*LOC_Os10g36924*), encodes a boric acid channel. It plays an important role in panicle development by regulating ROS homeostasis and SA biosynthesis.

## Materials and Methods

### Plant Materials and Growing Conditions

The rice *asa* mutant was a spontaneous mutant in line Q179, derived from the F_4_ progenies of a cross between *BobaiB* and *RW11*. It was crossed with *Nipponbare* [the wild-type (WT) plant], and the resultant F_1_ plants were selfed to produce F_2_ seeds for the mapping population. To allow fine mapping, first, a recessive individual from F_2_ exhibiting apical spikelet abortion was selected to successively backcross with the recurrent parent *Nipponbare* to construct the BC_2_F_1_ population; the BC_2_F_2_ population was derived from a BC_2_F_1_ individual in which the region around *OsASA* was heterozygous, and almost all other regions were homozygous. Meanwhile, to evaluate the agronomic traits, an *asa* recessive individual from the BC_2_F_2_ population whose agronomic traits were similar to the recurrent parent was crossed with *Nipponbare* to produce the BC_3_F_2_ population.

The F_2_, BC_2_F_1_, BC_2_F_2_, and BC_3_F_2_ populations were planted in the experimental field of the Rice Research Institute, Fujian Academy of Agricultural Sciences in Fuzhou, Fujian Province, China. Germinated seeds were sown in seed beds in mid-May, and 25-day-old seedlings were transplanted to the field. There was 20 cm between each plant in each row, and the rows were 20 cm apart. Field management, including irrigation, fertilizer application, and pest control, essentially followed normal agricultural practices. Additionally, transgenic plants were grown in pots in a greenhouse under standard growth conditions.

### Measurement of Agronomic Traits

To explore the morphological characteristics of the *asa* mutant with apical spikelet degeneration, the BC_3_F_2_ population was used to measure agronomic traits. This population was divided into three subpopulations: *asa* mutant (asa/asa), heterozygous (asa/Asa), and WT (Asa/Asa) populations by screening using the insertion–deletion (InDel) markers ID17. The agronomic traits (including plant height, panicles per plant, and panicle length) of the three plant types were assessed at the mature stage. Plant height was measured from the soil surface to the panicle tip of the main tiller (excluding the awn); the number of panicles per plant was evaluated by counting the panicle number of each plant; panicle length was scored from the neck node up to the tip of the last spikelet; each variable was averaged over 12 plants. Source-size traits, including flag leaf width and flag leaf length, were also measured at 5 days after heading.

### Microscopic Observation

For scanning electron microscopy (SEM) examination of young spikelets and pollen, samples were fixed in 2.5% (w/v) glutaraldehyde [in 0.1 M phosphate-buffered saline (PBS)] for 2 h and then washed three times with 0.1 M PBS. Following dehydration using an ethanol series at 4°C, the samples were subjected to critical-point drying, coated with palladium-gold using a sputter-coater, and observed under a scanning electron microscope (JSM-6390LV; JEOL).

Additionally, spikelets at various developmental stages were fixed in 2.5% (w/v) glutaraldehyde solution, post-fixed in 1% OsO_4_ solution, dehydrated using an acetone series, embedded in Spurr resin, sectioned, and stained with 0.1% toluidine blue. The spikelet sections were observed with a light microscope (DM4B; Leica) and a transmission electron microscope (HT-7800; Hitachi).

### Map-Based Cloning

Using the bulked segregant analysis (BSA) method, polymorphic markers were employed to analyze the mutant and WT DNA pools. Three simple sequence repeat (SSR) markers (RM25756, RM171, and RM147) co-segregated with the *OsASA* locus. A total of 169 F_2_ recessive individuals were used for the linkage analysis. The *OsASA* locus was located on chromosome 10 between RM25756 and RM171. Further markers with polymorphisms between the two DNA pools were then used to assess the 169 F_2_ recessive individuals and thereby narrow down the *OsASA* locus to the region between the InDel markers ID14 and ID16. For fine mapping and phenotyping, approximately 4500 individuals in the BC_2_F_2_ population were assessed. Among them, 978 *OsASA* recessive individuals were selected to screen for recombinants among InDel markers within the ID14–ID16 interval based on rough mapping of the F_2_ population. The recombinants were used for fine mapping, with newly developed markers between ID14 and ID16 being used to narrow down the *OsASA* locus to a 10.05-kb region between markers ID36 and ID16. Primers for the InDel markers are listed in Supplementary Table S1.

### RNA Isolation and Real-Time (RT)-PCR Analysis

Total RNA was isolated from the WT and *asa* mutant plants using an Eastep Universal RNA Extraction Kit (Promega). First-strand cDNA was synthesized by reverse transcription using an M-MLV First Strand Kit (Invitrogen). Quantitative RT-PCR was performed using SYBR Premix Ex Taq II (TaKaRa) on an ABI Step One Real-Time PCR system (Applied Biosystems). The rice *UBIQUITIN* gene was used as an endogenous control. All primers for RT-PCR are listed in Supplementary Table S2. The comparative threshold cycle (CT) method was used to analyze the relative expression levels.

### Plasmid Construction and Transformation of WT Plants

To analyze the expression pattern of the *OsASA* gene, a 2077-bp promoter fragment was amplified and then cloned into the binary vector pCXGUS-P, to generate the *pOsASA*:GUS construct. To create an *OsASA* overexpression vector, the full-length coding sequence of *OsASA* was amplified from the *Nipponbare* genome and then cloned into the binary vector pCXUN-FLAG by TA cloning. This created the *pUbi*:OsASA-FLAG plasmid, which was introduced into the *asa* mutant. To create the clustered regularly interspaced short palindromic repeats (CRISPR)-*OsASA* (*cr*-*OsASA*) transgenic plants that had the *OsASA* gene knocked out, a single guide RNA (sgRNA) sequence was designed using the CRISPR-PLANT website^1^. Primers containing the *Bsa*I digestion site were ligated into pU3-sgRNA, and the sgRNA fragment was then cloned into the binary vector pH-Ubi-cas9-7 using the LR recombination reaction. All transgenic rice plants were generated using *Agrobacterium*-mediated transformation of rice calli. All primers for plasmid construction are listed in Supplementary Table S2.

### β-Glucuronidase (GUS) Staining Assay

For the GUS staining assay, tissues were collected at various developmental stages, and the staining was performed as previously described (Jefferson, 1987). Images were captured using a stereoscope or a camera.

### Boron Treatment of Rice Seedlings

The seeds of the WT, *asa* mutant, and *cr*-*OsASA* (*OsASA*-knockout) transgenic plants were soaked and sowed in 96-well plates. They were grown in an incubator at 30°C in the light and at 25°C in the dark, with 10/14-h light/dark cycles. After 1 week, the seedlings were moved to 24-well plates, and the hydroponic culture solution was changed every 2 days. After 3 weeks of hydroponic culture, the seedlings were used for assessment of plant height, root length, and shoot and root fresh weight, along with assessing the SA level and the expression of *ISOCHORISMATE SYNTHASE 1* (*OsICS1*) and nine *PHENYLALANINE AMMONIA-LYASE* (*OsPAL*) genes. The hydroponic culture solution was prepared as described previously (Fukuda et al., 2004), with three boron concentrations (0, 15, and 150 μM) being used for each of the three types of plants (WT, *asa* mutant, and *cr*-*OsASA*).

### DAB Staining for H_2_O_2_

Spikelets collected at various developmental stages were incubated at 25°C with 1 mg/ml 3,3’-diaminobenzidine (DAB) plus 0.05% Tween-20, and they were subjected to vacuum infiltration for 10–15 min. After 12–24 h, destaining buffer (ethanol:acetic acid:glycerol = 3:1:1) was added. Images were captured using a stereoscope.

### Quantitative H_2_O_2_ Assays

The concentration of H_2_O_2_ in panicles was quantitatively determined using an H_2_O_2_ assay kit (Beyotime) according to the manufacturer’s instructions. Absorbance was measured with a spectrometer at a wavelength of 560 nm.

### Hormone Profiling

Hormones (including SA) were extracted as previously described. First, 100 mg of panicles was mixed with 1 ml of 80% methanol with internal standards (250 pg [^2^H_6_] ABA for ABA, SA, SAG, and JA; 250 pg [^2^H_5_] IAA for IAA and JA-ILE). Extraction was performed twice using a laboratory rotator for 2 h at 4°C. After 10 min of centrifugation at 18,000 *g* and 4°C, the supernatant was collected and dried using nitrogen gas. The pellet was then dissolved in 300 μl of 30% methanol. The hormones (including SA) were separated using a C18 column and analyzed using a triple quadrupole mass spectrometer as previously described (Šimura et al., 2018).

### RNA Sequencing

Young panicles of about 3 cm in length were collected from the WT and *asa* mutant plants to compare global gene expression changes between the WT and *asa* mutant. RNA extraction and RNA-seq analysis were performed by BioMarker (Qingdao, China), with three biological replicates.

## Results

### *asa* Mutant Exhibits an Apical Panicle Degeneration Phenotype

**FIGURE 1:**
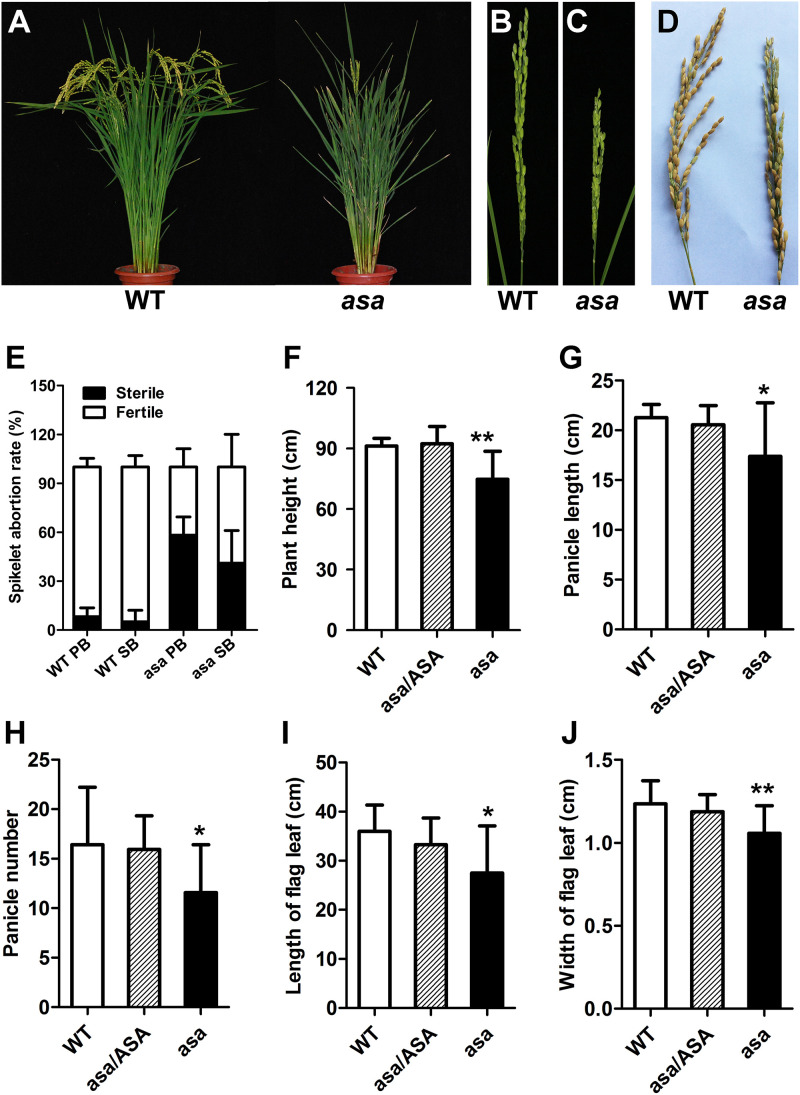
Phenotypic characterization of the *asa* mutant. **(A)** Phenotypic comparison between mature wild-type (WT) and *asa* mutant. Immature panicles phenotypic comparison between WT **(B)** and *asa* mutant **(C)**. **(D)** Mature panicles phenotypic comparison between WT and *asa* mutant. **(E)** Statistics of seed setting rate of WT and *asa* mutant. PB, primary branch; SB, secondary branch. Comparison of plant height **(F)**, panicle length **(G)**, panicle number **(H)**, length of flag leaf **(I)**, and width of flag leaf **(J)** between WT, hybrid type (*asa*/*ASA*), and *asa* mutant. Values are mean ± SD (*n* = 12); asterisks indicate significant differences (*P < 0.05, **P < 0.01) according to the Student’s *t*-test compared with the WT.

The rice *asa* mutant involved a spontaneous mutation found in line Q179, derived from the F_4_ offspring of a cross between *Bobai B* (*O. sativa* L. ssp. *indica* cv) and *RW11* (*O. sativa* L. ssp. *indica* cv) under natural conditions. Compared to WT plants, the *asa* mutants showed severe apical panicle degeneration at the heading stage; the apical spikelets exhibited various degrees of degeneration, while the middle and bottom of each panicle developed normally (Figures 1A–D and Supplementary Figure S1). The abortion rates for the primary and secondary branches in the *asa* mutants were about 55 and 40%, respectively, while scarcely any degenerated spikelets were observed in the WT plants (Figure 1E). In addition, the plant height was lower for the *asa* mutants than the heterozygous and WT plants (Figure 1F). Moreover, the panicle length and panicle number decreased significantly in the *asa* mutants, while these agronomic traits displayed no difference between the heterozygous and WT plants (Figures 1G,H). The flag leaf length and width were also obviously reduced in the *asa* mutants (Figures 1I,J). Thus, the *asa* mutant exhibits a pleiotropic phenotype, with severe apical panicle degeneration, and this significantly reduced the grain yield.

### *asa* Mutant Displays Defect in Spikelets

**FIGURE 2:**
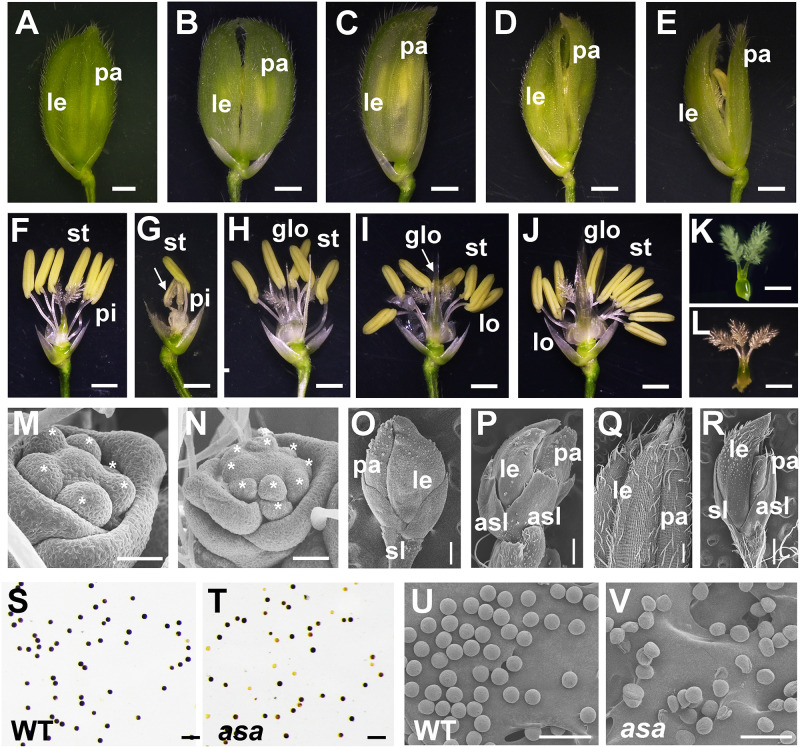
Spikelet morphology of wild-type (WT) and *asa* mutant. **(A–E)** Spikelet morphology of WT and *asa* mutant. The WT **(A)**, different phenotype types of *asa* mutants **(B–E)**. Bars = 1 mm. **(F–J)** Anther and pistil phenotype of WT and *asa* mutants. The WT **(F)**, different phenotype types of *asa* mutants **(G–J)**. Bars = 1 mm. Stigma morphology of WT **(K)** and *asa* mutant **(L)**. Bars = 1 mm. **(M,N)** Spikelet meristems at spikelet specification developmental stage. The WT **(M)** and *asa* mutant **(N)**. Stars mark the stamen primordia. Bars = 50 μm. **(O,P)** Spikelet meristems at stamen and pistil primordia differentiation stage. The WT **(O)** and *asa* mutant **(P)**. Bars = 200 μm. **(Q,R)** Epidermal surface of spikelets. The WT **(Q)**, *asa* mutant **(R)**. Bars = 500 μm. Potassium iodide staining of mature pollen grains of apical spikelets in WT **(S)** and *asa* mutant **(T)**. Bars = 100 μm. Scanning electron microscopy (SEM) observation of mature pollen grains of apical spikelets in WT **(U)** and *asa* mutant **(V)**. Bars = 100 μm. le, lemma; pa, palea; pi, pistil; st, stamen; lo, lodicule; glo, glume-like organ; asl, abnormal sterile lemmas.

A mature WT rice spikelet consists of a pair of glumes, a pair of sterile lemmas, two lodicules, six stamens, and one pistil with a bifurcated stigma (Figures 2A,F). Compared to the WT plants, the *asa* apical spikelets were defective, involving hooked or enlarged lemmas and palea (Figures 2B–E), twisted or whitish anthers with indefinite numbers, glume-like lodicules, and three-forked stigmas (Figures 2G–L).

To characterize the differences in spikelet meristem development, we used SEM to examine the panicle primordia of the WT and *asa* mutant plants at various developmental stages. At the spikelet specification developmental stage, in the WT plants, the lemma and palea primordia encapsulated six stamen of similar size and a single hemispherical pistil primordium (Figure 2M). In contrast, the *asa* mutants exhibited non-uniform stamen primordia, with varied numbers, irregularly surrounding the pistil primordium (Figure 2N). During the stamen and pistil primordia differentiation stage, the stamen and pistil primordia were enclosed by palea and lemmas and two sterile lemmas were located at the base of the spikelet in the WT plants (Figure 2O). However, in the *asa* mutants, there were several protrusions similar to palea or lemma primordia, and the sterile lemmas were drastically differentiated and larger than in the WT plants (Figure 2P). In the late stage of spikelet development, the WT palea and lemmas were wrapped around the floral organs, and the palea and lemma outer surfaces were composed of epidermal cells with neat papillary bulges; glume hairs and spiny tenuous hairs were located in the depressions between these papillary bulges (Figure 2Q). In contrast, the *asa* mutants had extra lemma-like organs and abnormal sterile lemmas, and the glume hairs were unevenly distributed on the surfaces (Figure 2R). Iodine–potassium iodide (I_2_-KI) staining showed that the pollen grains from *asa* apical spikelets were partly sterile compared to the WT plants (Figures 2S,T). SEM was used to further observe the pollen morphology. Compared to the normal pollen grains in the WT plants, the *asa* mutants had shrunken and irregularly shaped pollen grains (Figures 2U,V). These observations suggested that spikelet development was abnormal in the *asa* mutant.

### *asa* Mutant Exhibits Decreased Pollen Fertility

**FIGURE 3:**
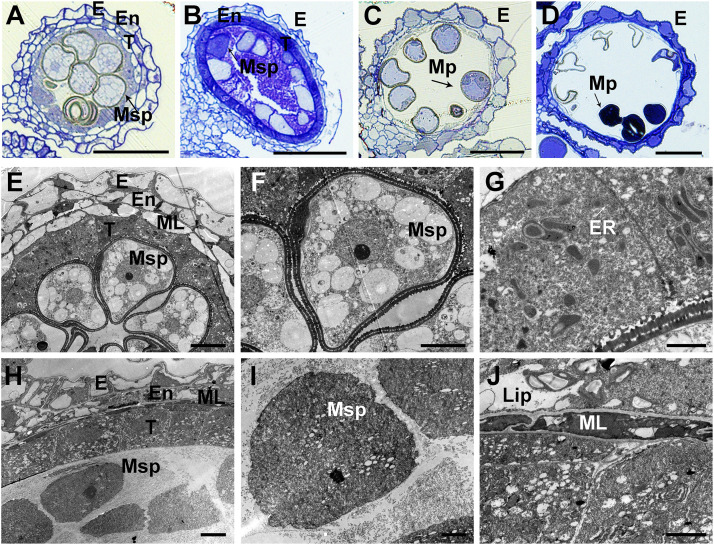
Transverse section and comparison between wild-type (WT) and *asa* anthers. Semithin sections of WT **(A,C)** and *asa* **(B,D)** anthers at middle young microspore stage, and mature pollen stage. Bars = 50 μm. **(E–J)** Transmission electron microscopy analysis of WT and *asa* anthers. Anthers of the WT **(E)** and *asa* **(H)**, young microspores in WT **(F)** and *asa* **(I)**, tapetal cells of WT **(G)** and *asa* **(J)**. Bars = 10 μm in **(E)**; 50 μm in **(F,H)**; and 2 μm in **(G,I,J)**. E, epidermis; En, endothecium; T, tapetum; Msp, microspores; MP, mature pollen; ML, middle layer; ER, endoplasmic reticulum; Lip, lipidosomes.

To further confirm the defects in the *asa* pollen grains, we prepared semithin sections of WT and *asa* anthers at various developmental stages. At the middle stages of microspore development in the WT plants, the spherical microspores exhibit multiple small vacuoles, with the tapetal cells becoming more vacuolated (Figure 3A). In the *asa* mutants, the pollen cavity accumulated unidentified substances, the microspores were irregularly shaped, and the tapetum was abnormal, without vacuolization (Figure 3B). Consequently, the WT plants had mature pollen grains, while some of the *asa* mutant microspores were degenerated, with a sickle shape and without starch accumulation (Figures 3C,D). To characterize the defects in the anther development, we used SEM examination of WT and *asa* anthers. Consistent with the findings of light microscopy of the transverse spikelet sections, the spherical microspores from WT plants formed multiple small vacuoles (Figures 3E,F). The *asa* pollen cavity accumulated unidentified substances, irregularly shaped microspores adhered to the inner side of the tapetum, and the tapetum was abnormally enlarged and thicker, without vacuolization (Figures 3H,I). The WT tapetum was condensed, with proliferated ER in the tapetal cells (Figure 3G). In contrast, almost no ER but abundant vacuoles and liposomes were observed in the *asa* tapetal cells (Figure 3J). These results indicated that *OsASA* may affect tapetal cell degradation and thereby control microspore formation.

### Map-Based Cloning and Characterization of *OsASA*

In the BC_2_F_2_ population, there were 383 WT and 87 mutant plants, with the frequencies fitting the expected Mendelian ratio (3:1) for single-locus segregation (χ^2^ = 1.07). We inferred that the *asa* trait was controlled by a single recessive gene.

To map the *OsASA* locus, 331 SSR markers on the 12 chromosomes (with a mean interval between markers < 5.0 cM) were selected to screen for polymorphic markers between the two parents, and for suspected markers linked to the *OsASA* locus, using the BSA method. We found that three SSR markers (RM25756, RM171, and RM147) co-segregated with the *OsASA* locus. Subsequently, 169 F_2_ recessive individuals were used for linkage analysis using the above three markers. The results showed that *OsASA* was located on chromosome 10 between SSR markers RM25756 and RM171, at distances of 1.2 and 4.7 cM, respectively. To narrow down the region, 29 InDel markers and five SSR markers were used. Of the 34 markers, 11 markers with polymorphisms between the mutant and WT DNA pools were used to map the 169 F_2_ recessive individuals, and linkage analysis showed that the *OsASA* locus was between InDel markers ID14 and ID16 at distances of 1.8 and 0.3 cM, respectively, while ID17 co-segregated with *OsASA.*

**FIGURE 4:**
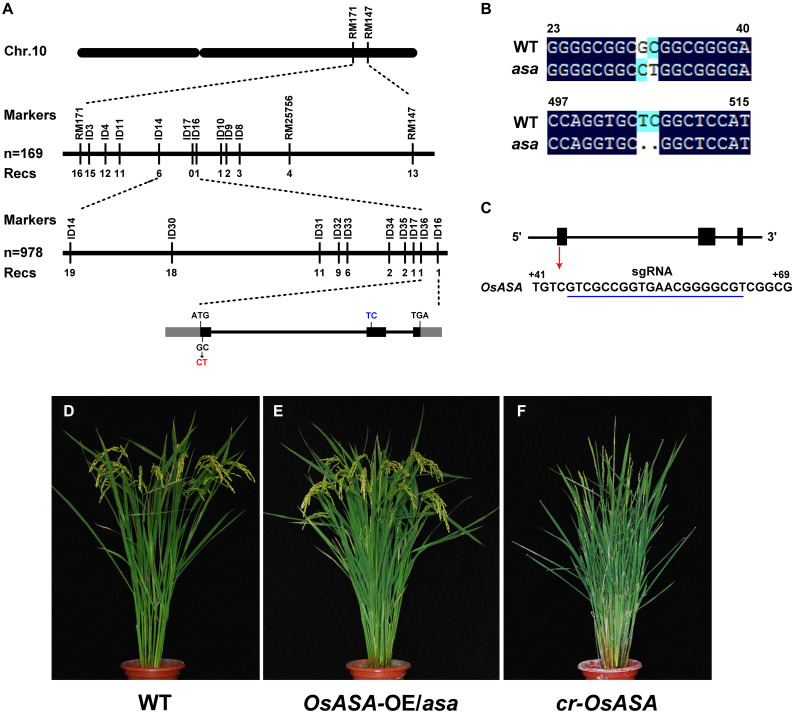
Map-based cloning of *asa.* **(A)** Fine mapping of *asa*. Molecular markers and numbers of recombinants are indicated above and below the bars, respectively. The gray and black boxes indicate untranslated regions and exons, respectively. **(B)** Alignment of the coding nuclear acid sequence of *OsASA* and *Osasa.* **(C)** Genomic structure of the *OsASA* gene. Black boxes indicate exons, and the sgRNA target sequence is underlined in blue. **(D–F)** Genetic confirmation of the *OsASA* gene. Wild type **(D)**, complemented with *OsASA* cDNA driven by the UBIQUTIN promoter introduced into *asa* mutant (*OsASA-OE*/*asa*) **(E)**, the knockout plant by CRISPR-Cas9 targets the first exon of *OsASA*(*cr*-*OsASA*) **(F)**.

For fine mapping of the *OsASA* QTL, an *OsASA* recessive individual was selected to successively backcross with the recurrent parent *Nipponbare* to construct the BC_2_F_1_ population. Approximately 4500 individuals in the BC_2_F_2_ population (which was derived from a BC_2_F_1_ individual with a *Nipponbare* genetic background and a heterozygous region around *OsASA*) were screened using the markers RM25756 and RM171. Among the 4500 individuals, 978 *OsASA* recessive individuals were selected to undergo screening for recombinants using the InDel markers ID14 and ID16. As a result, 19 recombinants were detected between ID14 and ID16, and then eight InDel markers within the two markers with polymorphisms were used to detect the 19 recombinants. Thus, the locus was delimited to a 10.05-kb interval between the InDel markers ID36 and ID16 (Figure 4A). The target region contains only one predicted gene, *LOC_Os10g36924*, based on the Rice Genome Annotation Project website^2^. Comparing the coding sequence of the *OsASA* genes revealed a two-nucleotide substitution of GC to CT in the first exon, and a two-nucleotide deletion in the second exon in the *asa* mutant (Figure 4B), which results in an amino acid substitution (Ala to Leu) and a frameshift. The *OsASA* gene comprises three exons and two introns, encoding a putative aquaporin NIP3;1 protein. Structural prediction showed that the OsASA protein has six transmembrane domains and two conserved Asn-Pro-Ala (NPA) motifs, while Osasa only contains two transmembrane domains and one NPA motifs (Supplementary Figure S2).

To confirm that the mutation in *LOC_Os10g36924* was responsible for the *asa* mutant phenotype, its full-length coding sequence driven by the rice *UBIQUITIN* promoter was introduced into an *asa* mutant background. All positive transformants (T_1_) displayed fertile spikelets, mimicking the WT phenotype (Figures 4D,E). This observation confirmed that mutation of *LOC_Os10g36924* (referred to as *OsASA*) underlies apical panicle abortion in the *asa* mutants. In addition, we transformed a CRISPR-Cas9 construct that targets the first exon of *OsASA* into the WT plant (*Nipponbare*) to knockout *OsASA* (Figure 4C), creating transgenic *cr*-*OsASA* plants. These plants exhibited the apical panicle abortion phenotype (Figure 4F). Collectively, these results demonstrated that the *OsASA* mutation was responsible for the *asa* mutant phenotype.

### *OsASA* Is Preferentially Expressed in Inflorescence

**FIGURE 5:**
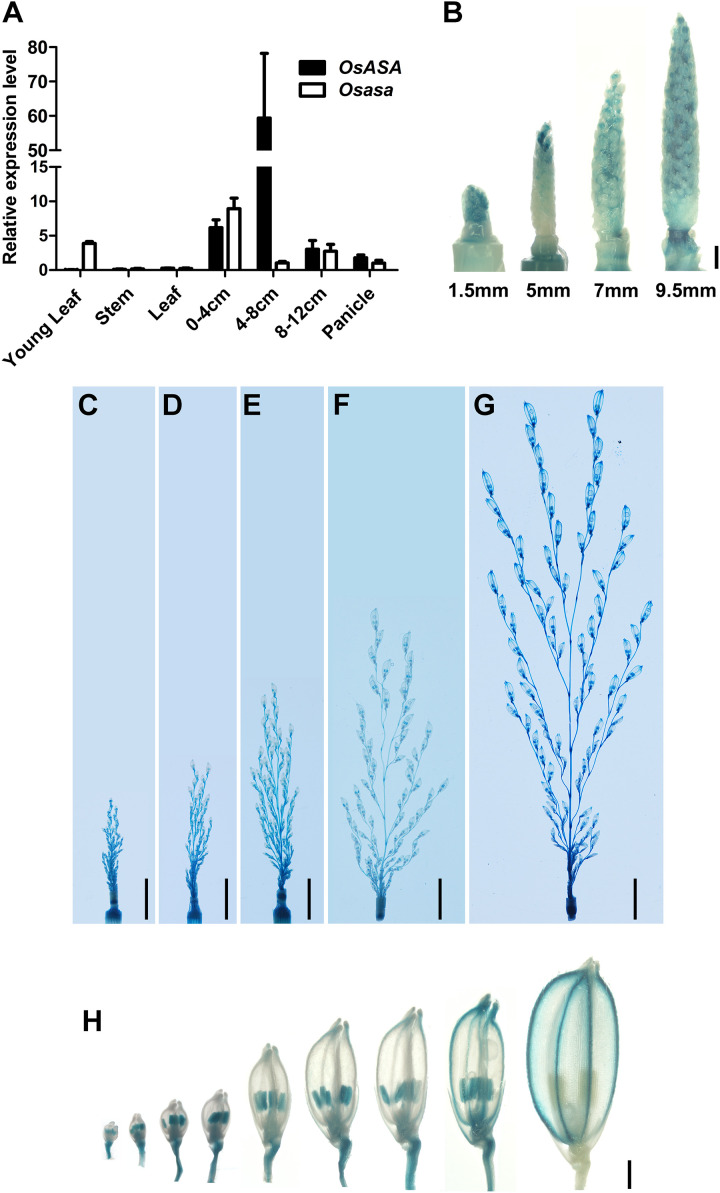
Expression pattern of *OsASA.* **(A)** Relative expression of *OsASA* in various tissues between wild-type and *asa* mutant. Rice *UBIQUITIN* gene was used as an internal control. 0–4, 4–8, and 8–12 cm present developing panicles at the 0–4, 4–8, and 8–12 cm stages. Values are mean ± SD. **(B)** Promoter activity of *OsASA* by GUS staining at early developmental stages of panicle. Different stages are shown as indicated by panicle length. Bars = 1 mm. **(C–G)** Promoter activity of *OsASA* by GUS staining at late developmental stages of panicles. Different stages are shown as indicated by panicle length, 2.5 cm **(C)**, 3.5 cm **(D)**, 5 cm **(E)**, 7.5 cm **(F)**, and 13 cm **(G)**. Bars = 1 cm. **(H)** Promoter activity of *OsASA* as shown by GUS staining in spikelets. Bars = 1 mm.

To elucidate the function of *OsASA*, we analyzed its expression pattern by subjecting the WT and *asa* mutant plants to qRT-PCR. In WT plants, *OsASA* expression was detected in all tissues analyzed, with relatively high expression in young panicles (0–4, 4–8, and 8–12 cm stages), especially the 4–8 cm stage, and lower expression in the young leaf, stem, and mature leaf (Figure 5A). However, the *asa* mutant had relatively low expression in 4–8 cm stage panicles and increased expression in the young leaf and 0–4 cm stage panicles (Figure 5A). The expression pattern of *OsASA* was further evaluated in transgenic plants harboring a *pOsASA*:GUS reporter vector, with the reporter gene driven by the 2077-bp promoter sequence of *OsASA*. GUS signals were observed in panicles at various developmental stages, with strong staining in young panicles and peduncles (1.5 mm, 5 mm, 7 mm, 9.5 mm, 2.5 cm, 3.5 cm, 5 cm, 7.5 cm, and 13 cm) (Figures 5B–G and Supplementary Figure S3). During spikelet development, strong GUS signals were observed in the anthers until the spikelets matured, and then the signals were only detected in the glumes (Figure 5H). Consistent with the *asa* phenotype, the *OsASA* expression was highest in the panicles. These results demonstrated that *OsASA* has a key role in panicle development.

### Transcriptome Analysis Highlights the Role of *OsASA*-Regulated ROS Homeostasis and SA Metabolism

**FIGURE 6:**
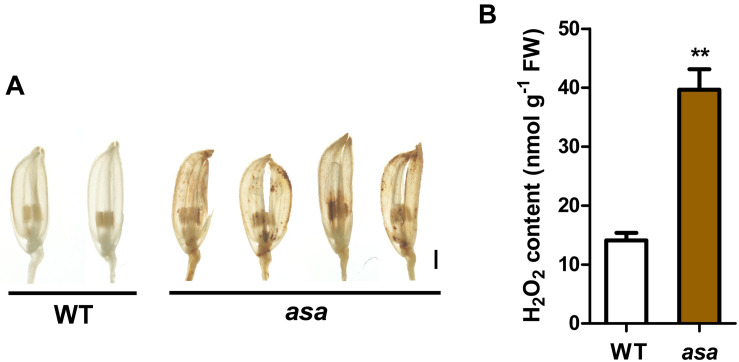
Reactive oxygen species accumulation in *asa.* **(A)** DAB staining of spikelets in wild-type and *asa* mutant. Bars = 1 mm. **(B)** Quantification of H_2_O_2_ from 3-cm stage panicles of the wild-type and *asa* mutant. Data are means ± SD (*n* = 3). Asterisks indicate significant difference from the wild type (**P < 0.01), as determined by Student’s *t-*test compared with the wild type.

We observed increased ROS levels in the degenerated *asa* apical spikelets by using DAB staining for H_2_O_2_ (which causes reddish brown precipitation) compared to that in the WT apical spikelets (Figure 6A). The DAB staining appeared in the *asa* apical panicles, being mainly distributed in several apical spikelets of the primary and secondary branches in the 3-cm panicles (Supplementary Figure S4). This suggested increased ROS accumulation at the early stage of *asa* panicle development. Quantitative measurements of H_2_O_2_ levels also showed that ROS levels were significantly higher in *asa* panicles than WT panicles (Figure 6B). These findings suggest a possible correlation between the ROS level and panicle degeneration.

**FIGURE 7:**
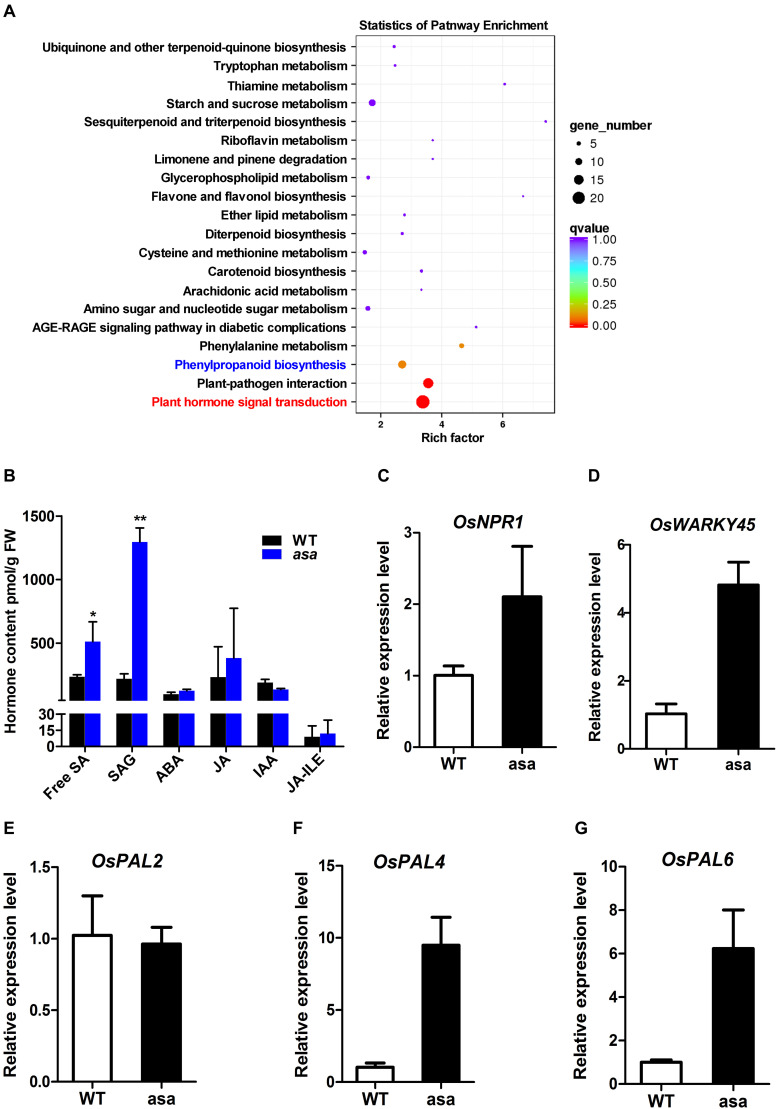
Transcriptome and hormone analysis of wild-type and *asa* mutant. **(A)** Kyoto Encyclopedia of Genes and Genomes (KEGG) enrichment of differentially expressed genes. **(B)** Hormone content of 3-cm young panicles in wild-type and *asa* mutant. SA, SAG, ABA, JA, IAA, and JA-ILE were measured. SA, salicylic acid; SAG, SA glucoside; ABA, abscisic acid; JA, jasmonic acid; IAA, indole acetic; JA-ILE, jasmonic acid-isoleucine. Data are means ± SD (*n* = 3), asterisks indicate significant difference from the wild type (*P < 0.05, **P < 0.01), as determined by Student’s *t-*test compared with the wild type. **(C–G)** RT-PCR analysis of candidate genes in the wild-type and *asa* mutant. Data are means ± SD (*n* = 3).

To further explore the molecular processes and functional pathways modulated by *OsASA* during panicle development, RNA-seq analysis of 3-cm panicles was used to compare global gene expression changes between the WT and *asa* mutant plants. There were 1505 differentially expressed genes (DEGs), with 655 down-regulated genes and 850 up-regulated genes in the *asa* mutants. Based on the annotated gene functions, 117 DEGs were involved in anther and pollen development (41 down-regulated and 76 up-regulated), 54 were involved in ROS homeostasis (22 down-regulated and 32 up-regulated), and 125 were related to SA metabolism (32 down-regulated and 93 up-regulated) (Supplementary Figure S5). The RNA-seq data were confirmed by performing qRT-PCR for five genes (Figures 7C–G). These results indicated that *OsASA* is involved in panicle development by regulating the ROS level and SA metabolism.

To further identify the pathways associated with the DEGs, Kyoto Encyclopedia of Genes and Genomes (KEGG) analysis was performed. This revealed that 135 (8.97%) DEGs were enriched in 73 KEGG pathways, including plant hormone signal transduction, plant–pathogen interaction, and phenylpropanoid biosynthesis (Figure 7A). To explore the possible phytohormone regulation of apical panicle abortion in the *asa* mutant, the endogenous levels of free SA, SA gluconside (SAG), indole-3-acetic acid (IAA), abscisic acid (ABA), and jasmonic acid (JA) in 3-cm stage panicles were measured. The free SA and SAG levels were significantly up-regulated in the *asa* mutants compared to the WT plants, while the IAA, ABA, JA, and JA-isoleucine (JA-ILE) levels did not differ (Figure 7B). These results suggested that SA is likely to play an important role in panicle development.

### *OsASA* Regulates SA Biosynthesis Under Boron-Deficient Conditions

**FIGURE 8:**
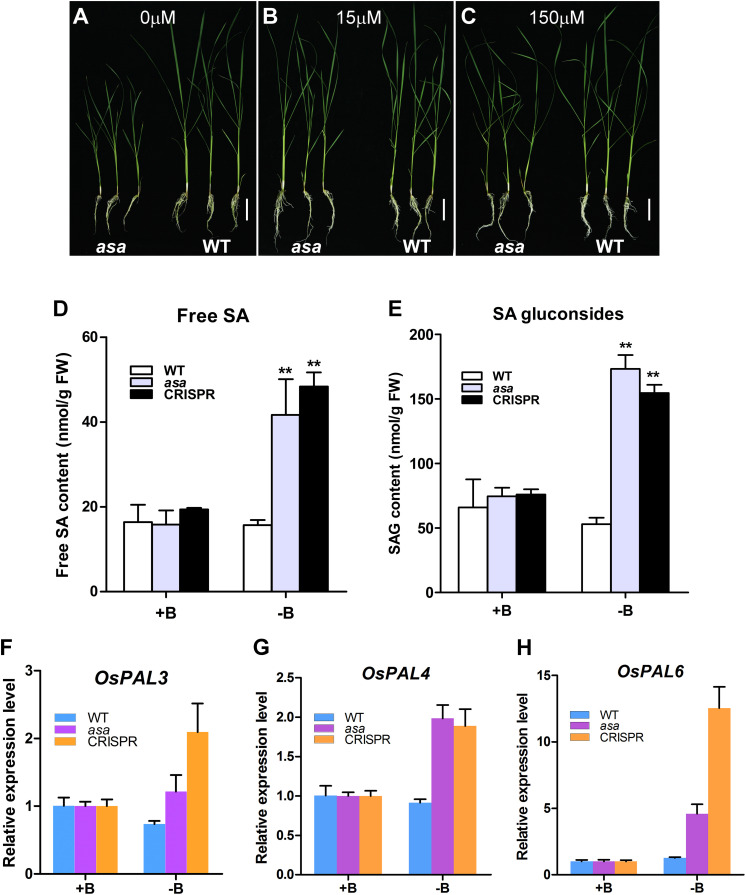
*asa* mutants were sensitive to boron and affect salicylic acid biosynthesis. Growth status of wild-type and *asa* mutant under 0 μM boron concentration **(A)**, 15 μM boron concentration **(B)**, and 150 μM boron concentration **(C)**. Bars = 5 cm. **(D,E)** Free salicylic acid and SA glucoside content of wild-type, *asa* mutant, and CRISPR-*OsASA* plants. Wild-type, *asa*, and CRISPR-*OsASA* plants were grown for 3 weeks in medium containing 15 μM boron (+B) or without boron (-B). Data are means ± SD (*n* = 3). Asterisks indicate significant difference from the wild type (**P < 0.01), as determined by Student’s *t-*test compared with the wild type. **(F–H)** Expression level of *OsPAL3*, *OsPAL4*, and *OsPAL6* in response to -B treatment. Wild-type, *asa*, and CRISPR-*OsASA* plants were grown for 3 weeks in medium containing 15 μM boron (+B) or without boron (-B). Data are means ± SD (*n* = 3).

To verify whether the *asa* mutants were defective in boron absorption, *asa* and WT seedlings were cultured for 3 weeks in hydroponic culture solution with various boron concentrations (0, 15, and 150 μM). In the 15 and 150 μM groups, the growth of *asa* mutants was indistinguishable from that of WT plants. However, in the boron-deficient group, the growth of *asa* mutants was seriously inhibited compared to that of WT plants (Figures 8A–C). In *asa* mutants under boron-deficient conditions, the plant height and shoot fresh weight clearly decreased compared to the values in the WT plants, while there were no significant differences in the length and fresh weight of roots (Supplementary Figures S6A–D). These findings indicated that *asa* mutants are sensitive to boron deficiency. Thus, *OsASA* may play an important role in boron transportation.

Salicylic acid level was significantly up-regulated in the young panicles of the *asa* mutant. To assess whether boron deficiency can affect SA metabolism, we measured the free SA and SAG levels and found that both of them were significantly up-regulated in *asa* and *cr*-*OsASA* plants under boron-deficient conditions (Figures 8D,E). We further examined the expression level of *OsICS1* and nine *OsPAL* genes, which are associated with SA biosynthesis, under boron-deficient conditions, and we found that *OsPAL3*, *OsPAL4*, and *OsPAL6* were up-regulated (Figures 8F–H), whereas *OsICS1*, *OsPAL1*, *OsPAL2*, *OsPAL5*, *OsPAL7*, *OsPAL8*, and *OsPAL9* showed no differences (Supplementary Figures S6E–K). Taken together, these results suggest that boron deficiency induces SA biosynthesis via the phenylalanine ammonia–lyase pathway.

## Discussion

### *OsASA* Is Involved in Panicle Development

Panicle degradation causes low seed setting and reduced grain yield. Several genes that are related to apical panicle degeneration, namely, *TUT1*, *OsALMT7*, *OsCIPK31*, *SPL6*, and *DPS1*, have been characterized (Bai et al., 2015; Heng et al., 2018; Peng et al., 2018; Wang Q. L. et al., 2018; Zafar et al., 2019). In this study, we isolated an apical panicle abortion mutant, the *asa* mutant, which exhibits apical panicle degeneration, abnormal spikelet development, and sterile pollen grains at the apical portion of panicles. *ASA* encodes a boric acid channel protein, identical to *NIP3;1* and *DTE1*, which plays an important role in regulating boron distribution in rice (Hanaoka et al., 2014; Liu et al., 2015; Shao et al., 2018). However, the mechanism of this gene in regulation rice reproductive development remains largely unclear. In this study, we discovered that the *asa* mutant harbors a mutation in *OsASA*, which encodes a boric acid channel (though the mechanism of this protein regarding the regulation of reproductive development in rice remains largely unclear). *OsASA* was preferentially expressed in the inflorescence. Additionally, in the *asa* mutant, genes involved in SA biosynthesis (*OsPAL3*, *OsPAL4*, and *OsPAL6*) were up-regulated, leading to increased SA levels and ROS accumulation, and thus affecting spikelet development and pollen fertility.

Panicle outgrowth in rice involves a rapid elongation process that requires higher rates of nutrient and energy supply (Heng et al., 2018). In particular, nitrogen nutrition and malate are required for normal panicle development (Fu et al., 2011; Heng et al., 2018). Boron is an essential micronutrient for the growth and development of vascular plants. Loss of function of boron influx channels or efflux transporters, such as in *bor1*, *nip5;1*, *nip6;1*, *rte1*, and *tls1* mutants, leads to plants with abnormal phenotypes under limited boron conditions, with reduced leaf and root growth, inflorescence defects, and sterility (Noguchi et al., 1997; Takano et al., 2006; Tanaka et al., 2008; Chatterjee et al., 2014; Durbak et al., 2014). Cereals such as rice and maize are especially sensitive to boron deficiency during reproductive development (Hu et al., 1996; Shorrocks, 1997; Lordkaew et al., 2011). The symptoms of boron deficiency first become visible in the growing tips of the plant, as boron is mainly required in developing tissues (Jiao et al., 2005; Krug et al., 2009; Lordkaew et al., 2011). We found that the *asa* mutant developed degenerated spikelets at the apical portion of the panicles (Figure 1). Meanwhile, it also exhibits similar phenotypes like retarded growth and increased number of tillers as *dte1* (Liu et al., 2015). Furthermore, spikelet meristem formation was defective in the *asa* mutant (Figure 2). *OsASA* was preferentially expressed in the inflorescence, peduncle, and young spikelet (Figure 5). Together, these results suggested that *OsASA* might support the preferential distribution of boron in inflorescence that is required to maintain panicle development.

### *OsASA* Plays a Role in Regulating ROS Homeostasis During Spikelet and Pollen Development

Reactive oxygen species are important signaling molecules that participate in a diverse range of biological processes (Mittler, 2017). ROS accumulation beyond a certain level often leads to apical panicle degeneration, defective anther development, and decreased pollen fertility (Hu et al., 2011; Bai et al., 2015; Zafar et al., 2019). ROS is an important trigger of tapetal programmed cell death, which is involved in tapetal degeneration during normal anther development (Li et al., 2006; Hu et al., 2011; Zhang and Yang, 2014). We identified many DEGs between the WT and *asa* mutant involved in the ROS network and anther development (Supplementary Figure S5), suggesting that *OsASA* may play a role in ROS homeostasis. Consistent with the *OsASA* expression profile analysis, higher H_2_O_2_ levels were detectable in the young panicles of the *asa* mutant, especially in the apical spikelets (Figure 6B). Increased DAB staining was observed in the apical panicles, mainly in multiple apical spikelets of the primary and secondary branches (Figure 6A and Supplementary Figure S4). These findings indicate that higher ROS accumulation is associated with the defective phenotype. In the *asa* mutant, the pollen cavity accumulated unidentified substances, the tapetum was abnormally enlarged and thicker, without vacuolization, and irregularly shape microspores adhered to the inner side of the tapetum, which may partly be the result of higher ROS levels during pollen development (Figure 3). Furthermore, the *asa* tapetal cells displayed features associated with oxidative stress, including intense cytoplasmic condensation and abundant vacuoles and liposomes (Figure 3). These findings indicate that the phenotype of *asa* mutant is accompanied by higher ROS accumulation in the apical spikelets, which causes the defective spikelets and sterile pollen development.

### *OsASA* Regulates SA Biosynthesis and Thereby Regulates Panicle Development

Salicylic acid is widely distributed in plants and plays an important role in the regulation of plant growth and development (Klessig and Malamy, 1994). It has been proposed that SA may also be involved in the regulation of spikelet degeneration under abiotic stress (Wang Z. Q. et al., 2018). Exogenous application of SA to heat-stressed plants significantly reduced spikelet degeneration and increased pollen viability (Zhang et al., 2017; Zhao et al., 2018). Other studies have also reported that SA plays important roles in regulating pollen viability, floret fertility, and the development of normal female flowers (Østergaard et al., 2002; Liu et al., 2019). It has been reported that phenylpropanoid can provide the key precursor for SA biosynthesis (Arrom and Munné-Bosch, 2012). In our study, 125 DEGs between the WT and *asa* mutant plants were involved in SA metabolism (Supplementary Figure S5). Additionally, the KEGG analysis revealed that biological processes related to phenylpropanoid biosynthesis were significantly enriched in the *asa* mutant (Figure 7A). Furthermore, SA accumulated in the young panicles of the *asa* mutant (Figure 7B). Under boron-deficient conditions, SA was significantly enriched in *asa* and *cr*-*OsASA* plants, and three genes (*OsPAL3*, *OsPAL4*, and *OsPAL6*) involved in SA biosynthesis were up-regulated (Figure 8). These observations suggest that SA accumulation suppresses apical spikelet development and is thus involved in spikelet degeneration.

In summary, our results showed that *OsASA*, which encodes a boric acid channel, regulates ROS homeostasis and SA biosynthesis, thus affecting spikelet development and pollen fertility.

## Data Availability Statement

The datasets presented in this study can be found in online repositories. The names of the repository/repositories and accession number(s) can be found below: https://www.ncbi.nlm.nih.gov/, PRJNA680625.

## Author Contributions

LC and MZ supervised the project and revised the article. LC, MZ, and DZ designed the experiments. DZ performed most of the experiments, analyzed the data, and wrote the article. The other authors assisted in experiments and analyzed the data. All authors contributed to the article and approved the submitted version.

## Conflict of Interest

The authors declare that the research was conducted in the absence of any commercial or financial relationships that could be construed as a potential conflict of interest.

## Footnotes

1 http://www.genome.arizona.edu/crispr/CRISPRsearch.html

2 http://rice.plantbiology.msu.edu/
