# Supplementary material for: APICAL SPIKELET ABORTION (ASA) Controls Apical Panicle Development in Rice by Regulating Salicylic Acid Biosynthesis
Source: Front Plant Sci. 2021 Feb 25;12:636877. doi: 10.3389/fpls.2021.636877 (PMC7947001; doi:10.3389/fpls.2021.636877)
Supplement: Supplementary file 1 [file Data_Sheet_1.docx]

Supplementary Figure

Figure S1. Comparison of young panicle in asa and the wild type, showing apical spikelet degeneration in asa mutant

Figure S2. Alignment of the amino acid sequences of OsASA in wild type and asa mutant

Figure S3. GUS staining of 1-cm length young panicles

Figure S4. DAB staining of young panicles

Figure S5. Expression patterns of genes involved in anther and pollen development, salicylic acid metabolism and ROS homeostasis

Figure S6. Growth defect of asa mutant and OsICS1, OsPAL genes expression level underboron-deficient conditions

Table S1. Markers used in fine mapping of the asa locus

Table S2. Primers used for plasmid construction and RT-PCR analysis

**Figure S1. Comparison of young panicle in wild type and *asa* mutant, showing apical spikelet degeneration in *asa* mutant.**

Developing panicles of the wild type (left) and *asa*(right), showing different stages as indicated by panicle length, from left to right: 1.5cm (A), 3.5cm (B), 6.5cm (C), 15cm (D). White arrows indicate the degenerating spikelets in *asa* mutant. Bars=1cm.

**Figure S2. Alignment of the amino acid sequences of OsASA in wild type (WT) and *asa* mutant.**

(A) Analyzing the transmembrane domain of OsASA protein by TMHMM.

(B) Protein structural comparison of OsASA and Osasa.

The red horizontal line represents the transmembrane domain, the green box represents the NPA domain, and M1-M6 represent the first to sixth transmembrane domain.

**Figure S3. GUS staining of 1-cm length young panicles.**

(A) GUS staining of the top spikelet from a 1-cm length young panicles branch. Bars=0.5mm.

(B) GUS staining of the bottom spikelet from a 1-cm length young panicles branch. Bars=0.5mm.

**Figure S4. DAB staining of young panicles.**

DAB staining of 3-cm length young panicles of wild type (A) and *asa* mutant (B). Bars=1cm.

**Figure S5. Expression patterns of genes involved in anther and pollen development, salicylic acid metabolism and ROS homeostasis.**

(A) Heat maps showing DEGs related to anther and pollen development.

(B) DEGs related to salicylic acid metabolism.

(C) DEGs related to ROS homeostasis.

**Figure S6. Growth defect of *asa* mutants and *OsICS1*, *OsPAL* genes expression level under boron-deficient conditions.**

(A-D) Physiological data statistics of wild type and *asa* mutant under different boron concentration, height of shoots (A), length of primary roots (B), fresh weight of shoots (C), fresh weight of roots (D). All data are presented as mean ±SD (*n*=6). Asterisks indicate signiﬁcant difference from the wild type (**P<0.01), as determined by Student’s *t* test compared with the wild type.

(E-K) Relative expression of *OsICS1*, *OsPAL* genes in response to -B treatment. Wild type, *asa* and CRISPR-*OsASA* plants were grown for 3 weeks in medium containing 15μM boron (+B) or without boron (-B). Data are means ±SD (*n*=3).
