# Supplementary material for: APICAL SPIKELET ABORTION (ASA) Controls Apical Panicle Development in Rice by Regulating Salicylic Acid Biosynthesis
Source: Front Plant Sci. 2021 Feb 25;12:636877. doi: 10.3389/fpls.2021.636877 (PMC7947001; doi:10.3389/fpls.2021.636877)
Supplement: Supplementary file 2 [file Data_Sheet_2.docx]

**Table S1. Markers used in fine mapping of the *asa* locus**

| **Marker** | **Forward primers** | **Reverse primers** |
| --- | --- | --- |
| ID14 | TGGTTCACACACAAATCTGA | CGTGTCCAACATTTGACTG |
| ID30 | AGGTCCCTCAACTTGTCACT | TCGGCATCCTCTTATACAAA |
| ID31 | AGCGTCTAGGTGAACACTGA | GGTTTTAACGGATCCATCTT |
| ID32 | AGTTGGGAGGAGTAGGTGAG | TTGCTGCAAGATATGGAAGT |
| ID33 | GTCCCAAAATTCAGACGATA | AATCTGGAGAAGCTGGAAAC |
| ID34 | ATAAAAACTGCAGTGGCAAG | TCTCCAAAATATGCAGTGCT |
| ID35 | GACGGTAACAACATCCTTGA | GATGGATGCTGTCAAATGTT |
| ID17 | GAATACACAGGCAGAGATGC | CTGATAATGCGAGACTGCTC |
| ID36 | GCGTGTTTTCTTTGTAGACG | CATACCAAATTACCGTGGAA |
| ID16 | AATTCTCGAGGTCTCTCGTC | CGTTGTCCAATGTCACCTAT |
|  |  |  |

**Table S2**. **Primers used for plasmid construction and RT-PCR analysis**

| **Primer name** | **Primer sequence(5’-3’)** |
| --- | --- |
| **Complementation testOsASA-FLAG** | |
| OsASA-CDS-F | AATGGAGATGGCGGCGCCGAACG |
| OsASA-CDS-R | TCAGCGGCGGAAGCTGCGCTGG |
| **CRISPR/CAS9 construct** | |
| OsASA-sgRNA-BsaI-F | GGCAGTCGCCGGTGAACGGGGCGT |
| OsASA-sgRNA-BsaI-R | AAACACGCCCCGTTCACCGGCGAC |
| **GUS analysis** | |
| OsASA-Pr-F | CATAGGTTCAGGTTTCAGGCTGCTC |
| OsASA-Pr-R | CCGCCATCTCCATTAATATATCAAC |
| **Primers for RT-PCR** | |
| UBQ-F | GCACAAGCACAAGAAGGTGA |
| UBQ-R | CCAAAGAACAGGAGCCTACG |
| ASA-F | TCAACCTCCTCTTCGTCGTC |
| ASA-R | ATATCCACAGCTGCCGGTAG |
| NRP1-F | CTGGGTTCTGGTGCAAATCC |
| NRP1-R | AACCTCTTCCTCTTCTCCGC |
| WARKY45-F | CAATCGTCCGGGAATTCG |
| WARKY45-R | GCCTTTGGGTGCTTGGAGT |
| ICS1-F | CAGGCCTGTACCCGGAGAT |
| ICS1-R | GCCATTGCTGGTGTAGGTGG |
| PAL1-F | GCCATCATGGAGCACATCC |
| PAL1-R | GCGTACCTGTCCTGCTTCG |
| PAL2-F | AGCACGGCTTCTTCGAGTTG |
| PAL2-R | GTTGGCCTCAAAGAGCACG |
| PAL3-F | TGAGGCGAACATTCTTGCTG |
| PAL3-R | GCTTGTGTGTCAGGTGGTCG |
| PAL4-F | GGACAGCTACGGTGTCACCA |
| PAL4-R | CGGCATTGAGGAATCTGATGA |
| PAL5-F | CATGGACAACACCCGCCT |
| PAL5-R | AGGTTTGACGTGAGCCCG |
| PAL6-F | CCACCTGACGCACAAGCTTA |
| PAL6-R | TCTTCGCCAGCATCATGTAG |
| PAL7-F | CGAGCAGCACAACCAGGAC |
| PAL7-R | CGAGGAAGGTGGAGGACATG |
| PAL8-F | CCCGAGCTTGGACTACGGT |
| PAL8-R | TCTGCACATGGTTGGTCACC |
| PAL9-F | CGAGCTTGGACTACGGTTTCA |
| PAL9-R | GGTCTGCACATGGTTGGTCAC |
