# Supplementary figures and images for: APICAL SPIKELET ABORTION (ASA) Controls Apical Panicle Development in Rice by Regulating Salicylic Acid Biosynthesis
Source: Front Plant Sci. 2021 Feb 25;12:636877. doi: 10.3389/fpls.2021.636877 (PMC7947001; doi:10.3389/fpls.2021.636877)

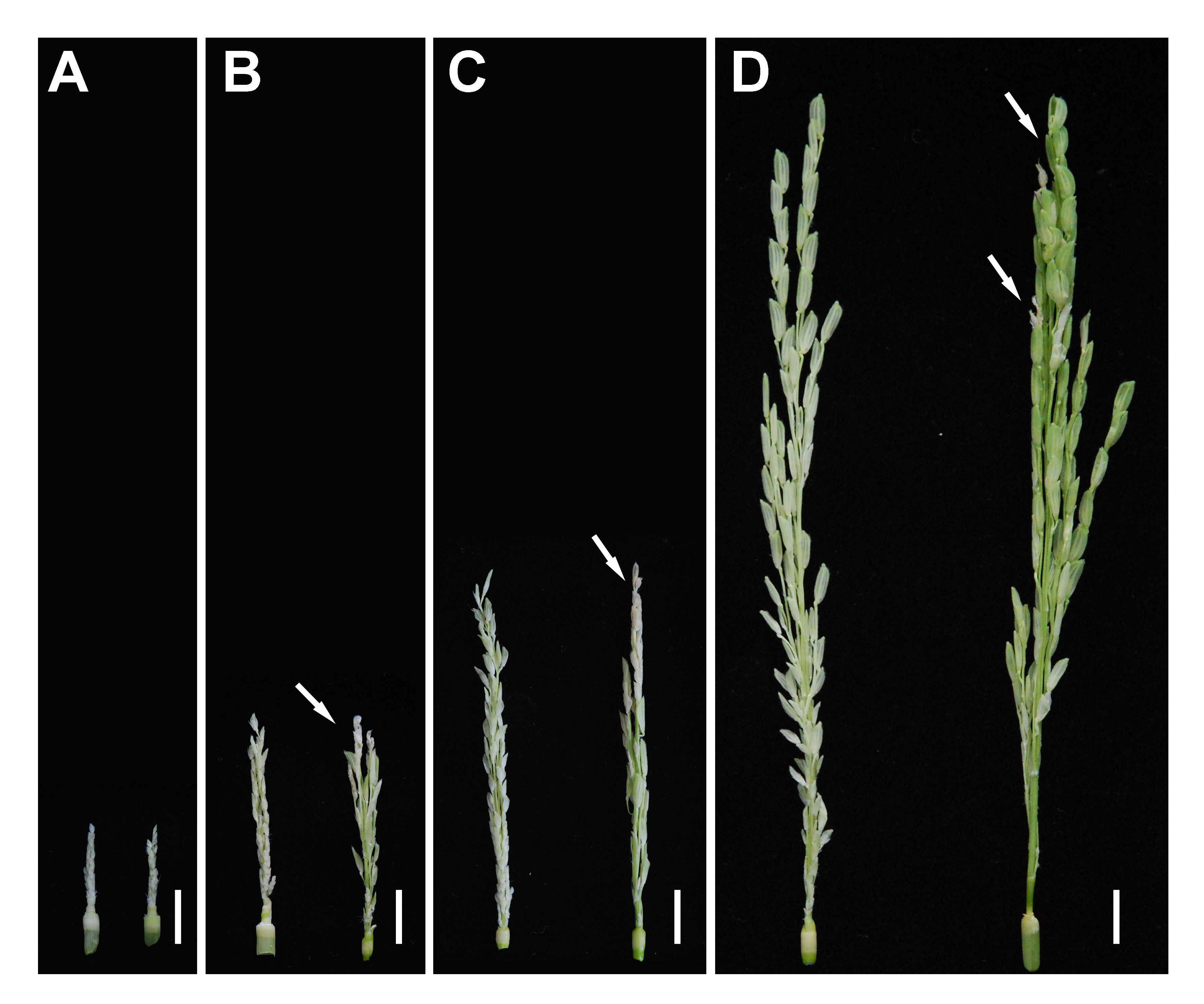

Supplement: Supplementary file 3 [file Image_1.TIF]

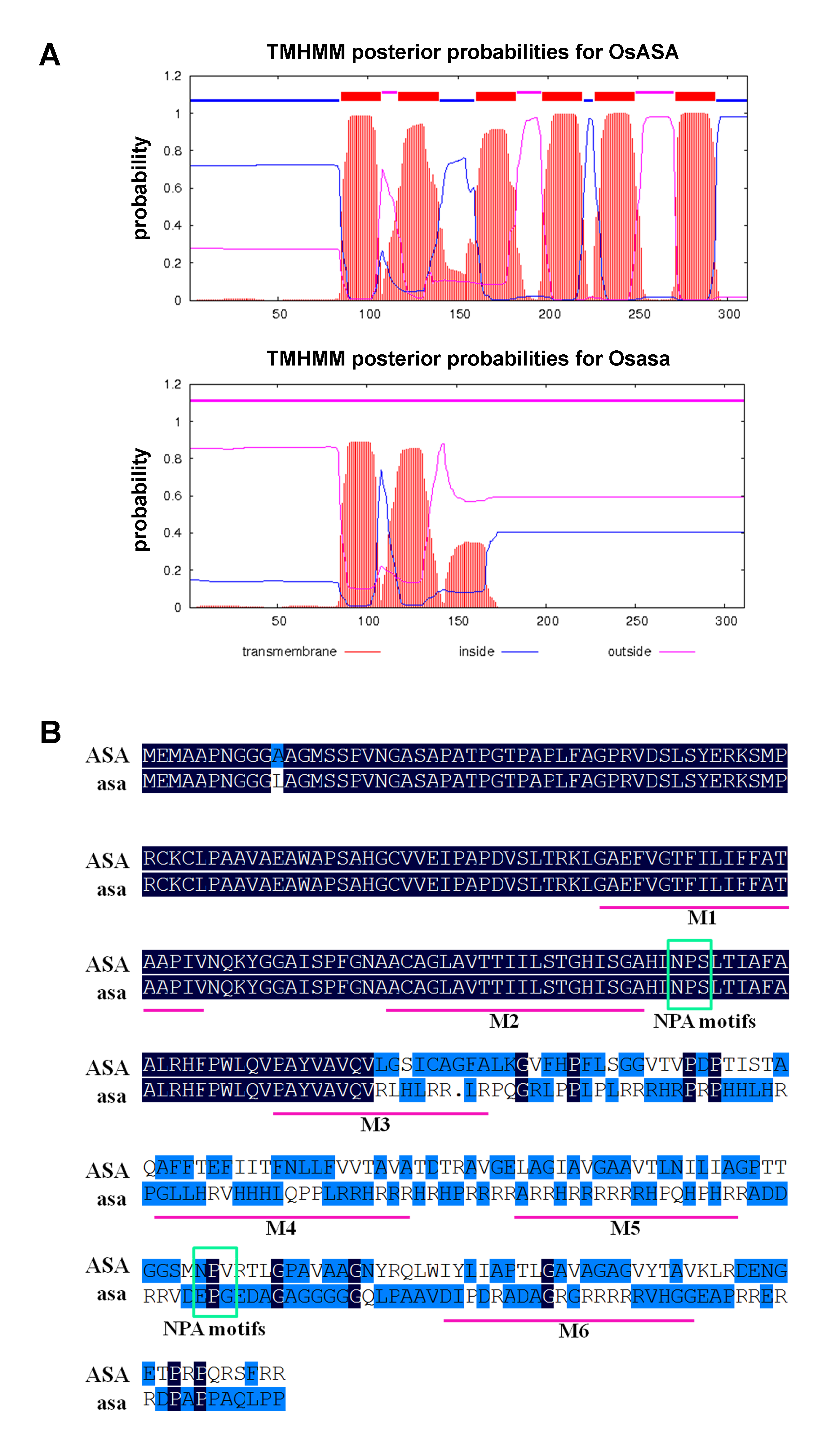

Supplement: Supplementary file 4 [file Image_2.TIF]

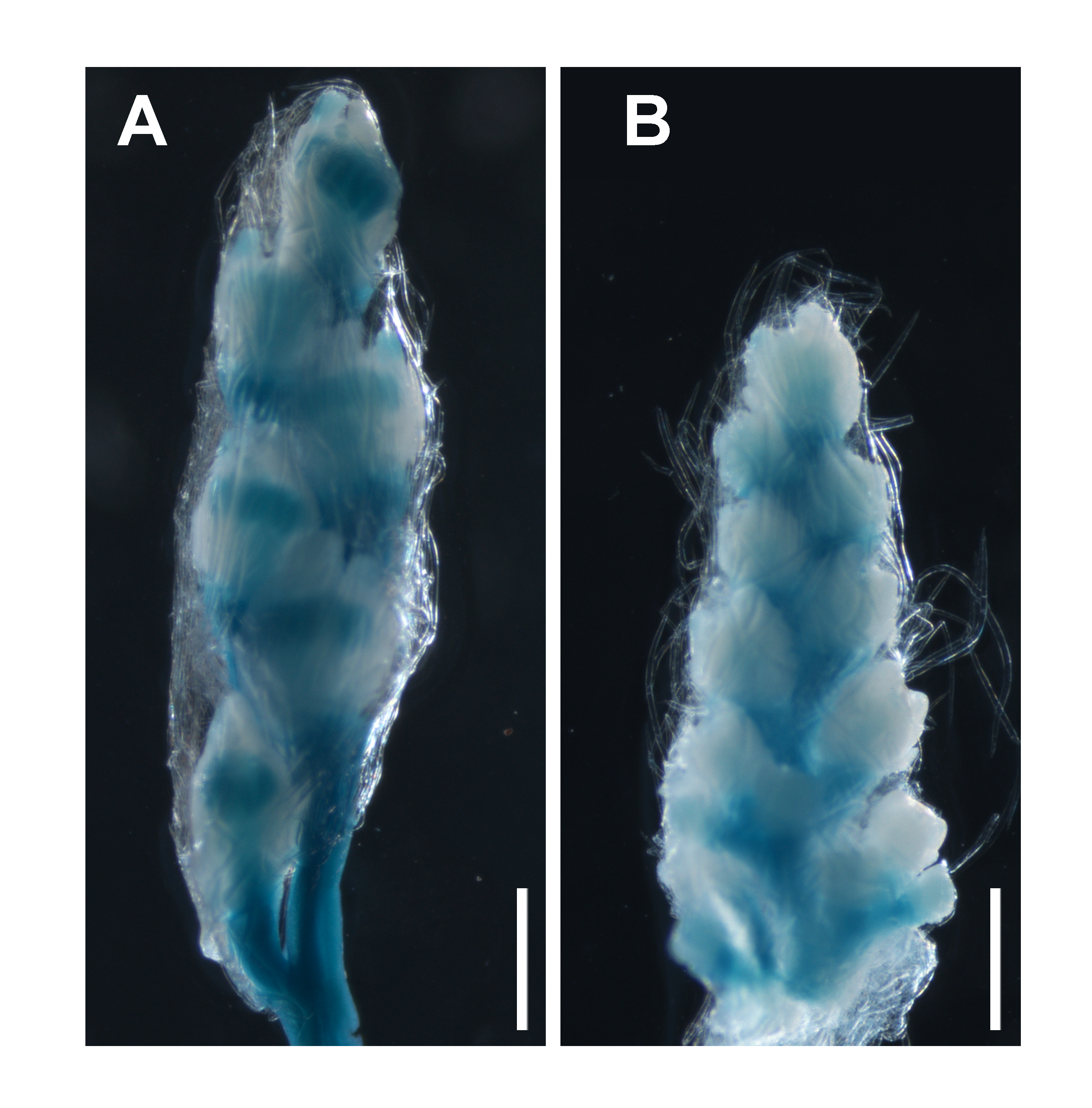

Supplement: Supplementary file 5 [file Image_3.TIF]

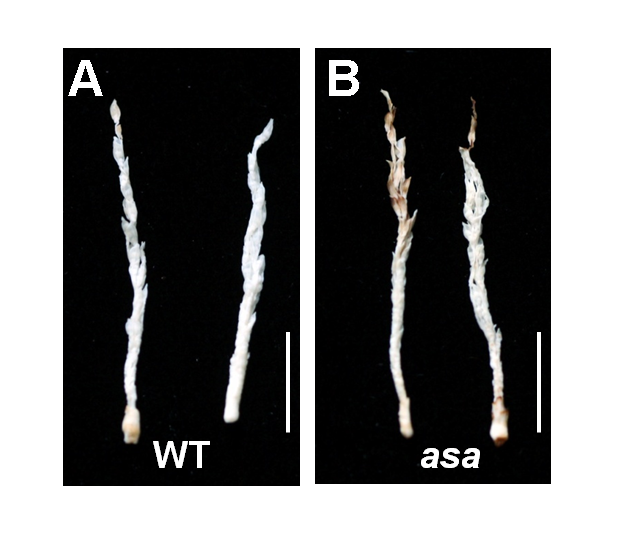

Supplement: Supplementary file 6 [file Image_4.TIF]

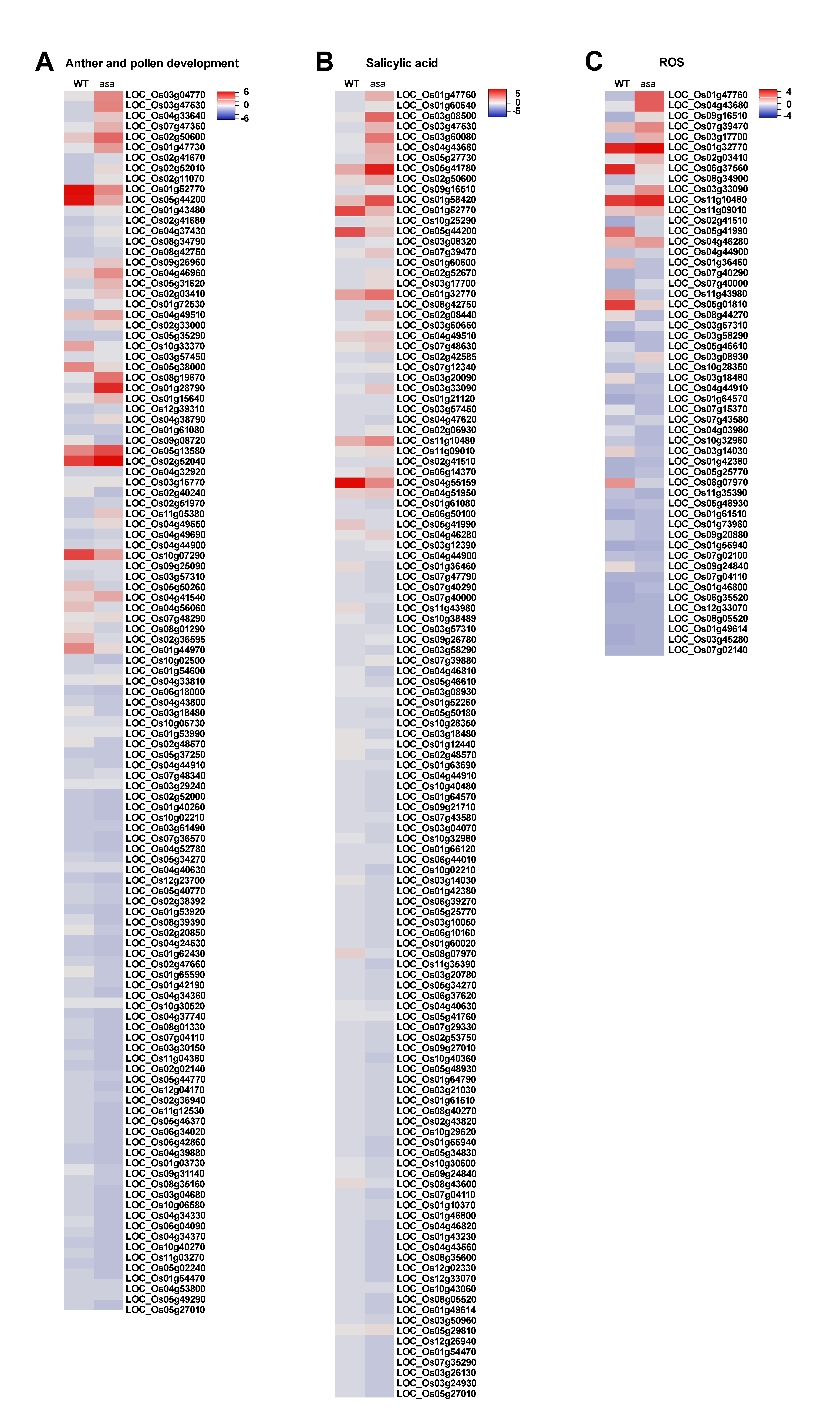

Supplement: Supplementary file 7 [file Image_5.TIF]

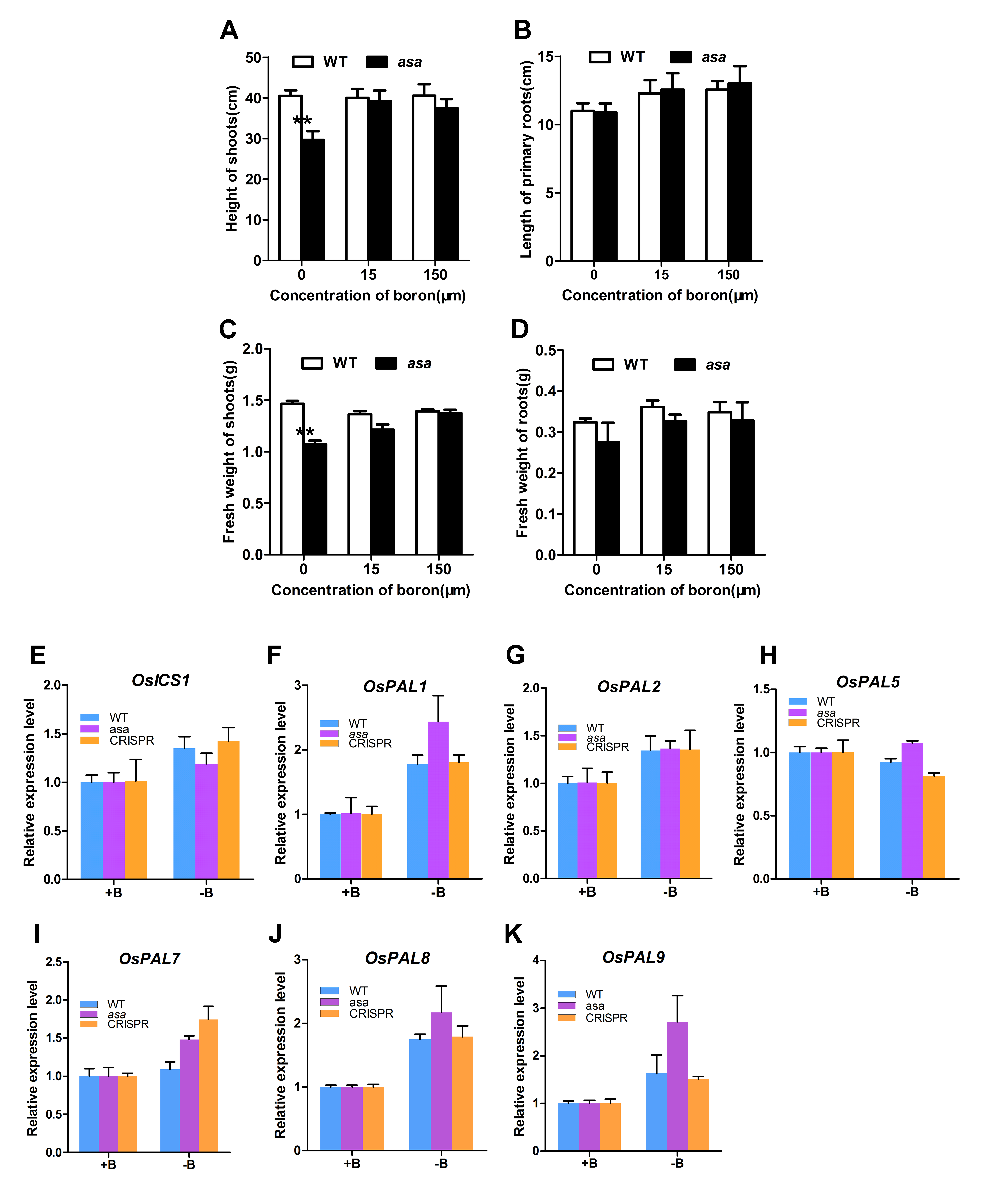

Supplement: Supplementary file 8 [file Image_6.TIF]
